# Habitat preference and diverse migration in threespine sticklebacks, *Gasterosteus aculeatus* and *G. nipponicus*

**DOI:** 10.1038/s41598-020-71400-4

**Published:** 2020-08-31

**Authors:** Takaomi Arai, Daisuke Ueno, Takefumi Kitamura, Akira Goto

**Affiliations:** 1 Faculty of Science, Universiti Brunei Darussalam, Jalan Tungku Link, Gadong, 1410 Brunei Darussalam; 2 Faculty of Fisheries and Marine, Universitas Airlangga, Surabaya, 60113 Indonesia; 3 Graduate School of Fisheries Sciences, Kitasato University, 160-4 Sanriku, Ofunato, Iwate 022-0101 Japan; 4 Graduate School of Fisheries Sciences, Hokkaido University, Hakodate, 041-8611 Japan

**Keywords:** Animal migration, Ichthyology, Ecology, Zoology, Ecology

## Abstract

Threespine sticklebacks of the genus *Gasterosteus*, are small teleost fish that are widely distributed across the northern hemisphere. The fish is believed to have two major types of life history, freshwater resident and anadromous; however little is known about their migration ecology. Comprehensive research on the migratory history, habitat use and relative composition of migratory types was conducted by analysing the otolith strontium and calcium concentrations collected in various environments of northern Japan. The present study first demonstrated that approximately 90% of morphologically anadromous sticklebacks had estuarine resident migration pattern, consistently living in brackish water and/or marine environments through their life cycle without any time spent in freshwater. The dominant occurrence of the estuarine resident was temporally and spatially consistent with their general migration ecology. The estuarine resident is thought to be the ancestral migrations of *G. aculeatus* and *G. nipponicus*, which thereafter gradually immigrated into freshwater habitats and settled in the anadromous form in both species and finally became the freshwater resident *G. aculeatus*. Thus, this study provides novel insights into the evolutionary migration of these fish, as well as a new discovery regarding the dominant migratory history and habitat use in threespine sticklebacks.

## Introduction

Threespine sticklebacks of the genus *Gasterosteus* are small teleost fish that are widely distributed throughout the Northern Hemisphere^1^. The ethology of the stickleback was enthusiastically studied in the twentieth century, in which its courtship and aggressive behaviours were characterized^2,3^. The species is a well-suited model for studying the genetic mechanisms underlying variations in migratory behaviours, since the species is adaptable to a wide range of environmental conditions. The life span of threespine sticklebacks varies from annual to 6 years depending on geographical locations and different habitats^1,4,5^. The fish are widely distributed in various aquatic environments such as freshwater, brackish water and seawater, and have been divided into an anadromous type and a freshwater resident (fluvial) type^1^. The freshwater type was found to have recolonized from anadromous populations after the last deglaciation approximately ~ 10,000–20,000 years ago^6–15^. Anadromous and freshwater resident stickleback populations are usually allopatric but are occasionally parapatric or sympatric^7,16–24^. Thus, their migratory ecology and behaviour throughout the life cycle are variable and complicated.

Threespine sticklebacks around the Japanese archipelago are found to occur in two species, *Gasterosteus aculeatus* and *G. nipponicus*^24,25^. *G. nipponicus* is thought to have diverged from an ancestral *G. aculeatus* and acquired unique traits during a period of isolation in the old Sea of Japan approximately two million years ago^24,25^. Hybrids between the two species were found at an extremely low ratio in the sympatric areas, suggesting that reproductive isolation exists^24,25^. The initial divergence started in allopatry, while competitive interactions in sympatry played a major role in the evolution of reproductive isolation and resource partitioning^21^. The two species differ in their life history pattern: *G. aculeatus* comprises freshwater resident, anadromous and estuarine residents, and *G. nipponicus* comprises anadromous and estuarine residents^24–27^. Compared with the extensive ethological, evolutionary, parasitological and toxicological research, little is known about the migration of threespine sticklebacks. Our previous studies examined several (five) specimens only in the each location^26,27^ and thus, the relative occurrence of each migratory type as well as the minute migratory history in *G. aculeatus* and *G. nipponicus* from wide distribution range remains temporally and spatially unclear. Individual migratory history data would provide fundamental knowledge about the migration ecology and behaviour in threespine sticklebacks.

Discrimination between anadromous and freshwater resident of *G. aculeatus* has been achieved by means of external morphological characteristics such as number of plates and their size distribution. Anadromous sticklebacks are a completely plated morph, whereas those in freshwater resident have a comparatively small number of plates (low plate morph)^17,23,28^. Their mean size is larger in anadromous compared to freshwater residents, and a bimodal size distribution was found to coexist in a habitat^3,7,29,30^. However, morphological characteristics are applicable to a limited area due to the local variations in the characteristics^3,29,31^. Novel research using otolith microchemistry has revealed a new migration pattern, estuarine resident, in threespine sticklebacks *G. aculeatus* and *G. nipponicus* that have never migrated into freshwater, but instead spent their entire life in brackish or seawater environments, co-existing with typical freshwater residents and those with anadromous life histories^26,27^. There were a number of discrepancies between the life history style estimated from morphology and the migration pattern estimated from otolith microchemistry, e.g., *G. aculeatus* morphologically classified as the freshwater resident type actually had an anadromous or estuarine resident life history^26,27^. The classical morphological observation is not necessarily reflecting the migratory types of sticklebacks.

The otolith is in the membranous labyrinth of the inner ear of teleost fishes. The otolith records various information that the fish has experienced previously, and this can be retrieved and reconstructed through the analysis of otolith microchemistry^32,33^. Sr can substitute for calcium in otolith aragonite due to its close proximity to Ca in ionic radius. The Sr:Ca ratio in the otolith is positively related to salinity, and hence, this ratio can be used as a tracer for reconstruction of the migratory history of diadromous fish^32,33^. Otolith microchemistry in threespine sticklebacks has revealed diverse migratory patterns in aquatic environments^26,27^ and it has elucidated the opportunistic anadromy with the occurrence of estuarine residents^26,27,34,35^. The otolith microchemistry has also revealed a new migration type in the ninespine stickleback, *Pungitius pungitius*; besides the two representative life history types, i.e., freshwater resident and estuarine resident (brackish water) life histories, the fish had an alternative anadromous life history^36–38^. In spite of the general acceptance of the use of otolith Sr:Ca ratios to describe the migration in diadromous fish species, the method has rarely been validated by controlled rearing studies under laboratory conditions. In the absence of controlled rearing studies in the threespine sticklebacks, our previous studies^26,27,34^ have relied on results from the other diadromous fish species. When the migratory history is studied using the otolith Sr and Sr:Ca ratios, the assessment of the applicability of the salinity response is indispensable.

In the present study, comprehensive research to understand the complex life history and migration strategy has been conducted by examining the otolith Sr concentration and Sr:Ca ratios in threespine sticklebacks, *G. aculeatus* and *G. nipponicus*, collected from various environments such as rivers, lakes, marshes, estuaries, tidal ponds, and coastal waters at 18 sites in northern Japan. The validation of the appropriateness of otolith Sr:Ca ratios for reconstructing the migratory history of fish has also been properly completed in the present study. The results provide temporal and spatial migration and habitat use details and reveal the diverse migration strategies between marine and freshwater habitats. Furthermore, the current research provides novel insights into the evolutionary history of their migrations, as well as a new discovery of the dominant migration and habitat use of threespine sticklebacks.

## Results

### Effects of salinity on otolith Sr:Ca ratios

The mean total lengths (TLs) in the four experimental salinities of 0, 10, 20 and 30 psu were 17.3 ± 0.5 (± SD) mm, 18.2 ± 1.3 mm, 18.6 ± 0.7 mm and 17.5 ± 0.8 mm, respectively. There were significant differences in TL among the four salinities (Kruskal–Wallis test, n = 40, H = 13.577, *p* < 0.005), and were found between 0 and 10 psu and 20 psu and between 20 and 30 psu (Mann Whitney-*U* test, df = 11–17, U = 6–20, *p* < 0.05–0.001). No significant differences were found for the other combinations (Mann Whitney-*U* test, df = 14–15, U = 33–42, *p* > 0.05).

The mean Sr and Ca concentrations in the rearing waters ranged from 0.03 ± 0.001 (± SD) mg l^-1^ (0 psu) to 8.12 ± 0.3 mg l^-1^ (30 psu) and from 4.97 ± 0.15 mg l^-1^ (0 psu) to 336 ± 28.3 mg l^-1^ (30 psu), respectively. The relationships between salinity (S) and Sr and Ca were Sr = 0.269S–1.17 (r^2^ = 0.996) and Ca = 10.99S–54.96 (r^2^ = 0.998), respectively. The Sr and Ca concentrations in the rearing water differed significantly among the four experimental salinities (Kruskal–Wallis test, n = 80, H = 74.967, *p* < 0.0001), and they both significantly increased with salinity (t-test, df = 80, *p* < 0.0001). The mean Sr:Ca ratios in the rearing waters ranged from 2.44 × 10^–3^ ± 0.08 × 10^–3^ (0 psu) to 9.64 × 10^–3^ ± 0.82 × 10^–3^ (30 psu). The relationship between salinity (S) and the Sr:Ca ratio in the water was water Sr:Ca ratio = 1.27S + 9.52 (r^2^ = 0.852) and the Sr:Ca ratio in the rearing water significantly increased with salinity (t-test, df = 80, *p* < 0.0001).

There were significant differences of the Sr:Ca ratio in the otoliths among the four experimental salinities (Kruskal–Wallis test, H = 22.813, *p* < 0.0001). The mean (± SD) Sr:Ca ratio in the otoliths significantly increased from 1.20 × 10^–3^ ± 0.98 × 10^–3^ in 0 psu to 4.30 × 10^–3^ ± 0.38 × 10^–3^ in 30 psu, and the Sr:Ca ratio in the otoliths was significantly correlated with the salinity (otolith Sr:Ca ratio = 0.09S + 2.13, r^2^ = 0.708; t-test, df = 39, *p* < 0.0001). These results suggest that the otolith Sr:Ca ratio in threespine sticklebacks is affected by ambient salinity.

The mean otolith Sr:Ca ratio in the wild sticklebacks that lived in an artificial freshwater pond was 1.31 × 10^–3^ ± 0.10 × 10^–3^ (± SD). No significant difference was found in the otolith Sr:Ca ratios between reared and wild sticklebacks in the freshwater environment (Mann Whitney-*U* test, df = 9, U = 47, *p* > 0.05). According to the linear regression between otolith Sr:Ca ratio and salinity and the otolith Sr:Ca values of freshwater in the reared and the wild sticklebacks and phase L (low Sr:Ca ratio) in the wild sticklebacks examined migratory history (see Migratory history), < 2.5 × 10^–3^ was used as an indicator of a freshwater environment (freshwater resident), distinct from a brackish or marine environment, in the present study. We also categorized the specimens into “anadromous” which had a transition point (TP) from phase-L (low Sr:Ca ratios < 2.5 × 10^–3^) to phase-H (high Sr:Ca ratios ≥ 2.5 × 10^–3^) along the line history transect and “estuarine resident” which showed constantly high Sr:Ca ratios (≥ 2.5 × 10^–3^) from the otolith core to the edge, followed in previous^26,27^ and present studies.

### Migratory history

**Figure 1:**
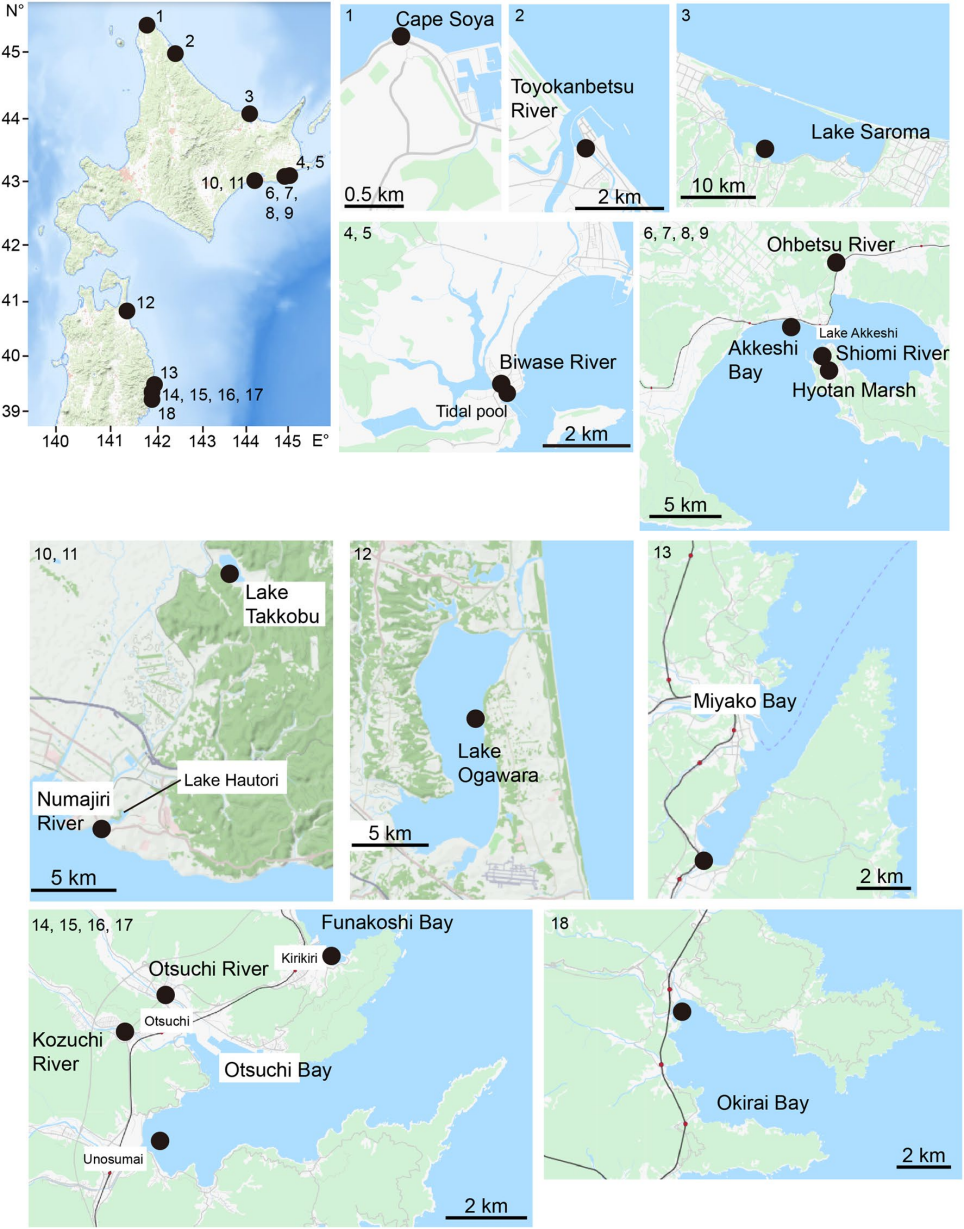
The locations where the threespine sticklebacks, *Gasterosteus aculeatus* and *G. nipponicus*, were collected. Map of sampling locations of the threespine sticklebacks, *G. aculeatus* and *G. nipponicus* in northern Japan. Numbers with the black dots on the map of northern Japan (upper left corner) correspond to each sampling site. 1, Cape Soya, 2, Toyokanbetsu River, 3, Lake Saroma, 4, Biwase River, 5, Biwase River tidal pool, 6, Hyotan Marsh, 7, Shiomi River, 8, Obetsu River, 9, Akkeshi Bay, 10, Numajiri River, 11, Lake Takkobu, 12, Lake Ogawara, 13, Miyako Bay, 14, Funakoshi Bay, 15, Otsuchi Bay, 16, Otsuchi River, 17, Kozuchi River, 18, Okirai Bay. The base map was downloaded from the USGS National Map Viewer (open access) at https://viewer.nationalmap.gov/viewer/ and from the OpenStreetMap (open access) at https://www.openstreetmap.org.

**Figure 2:**
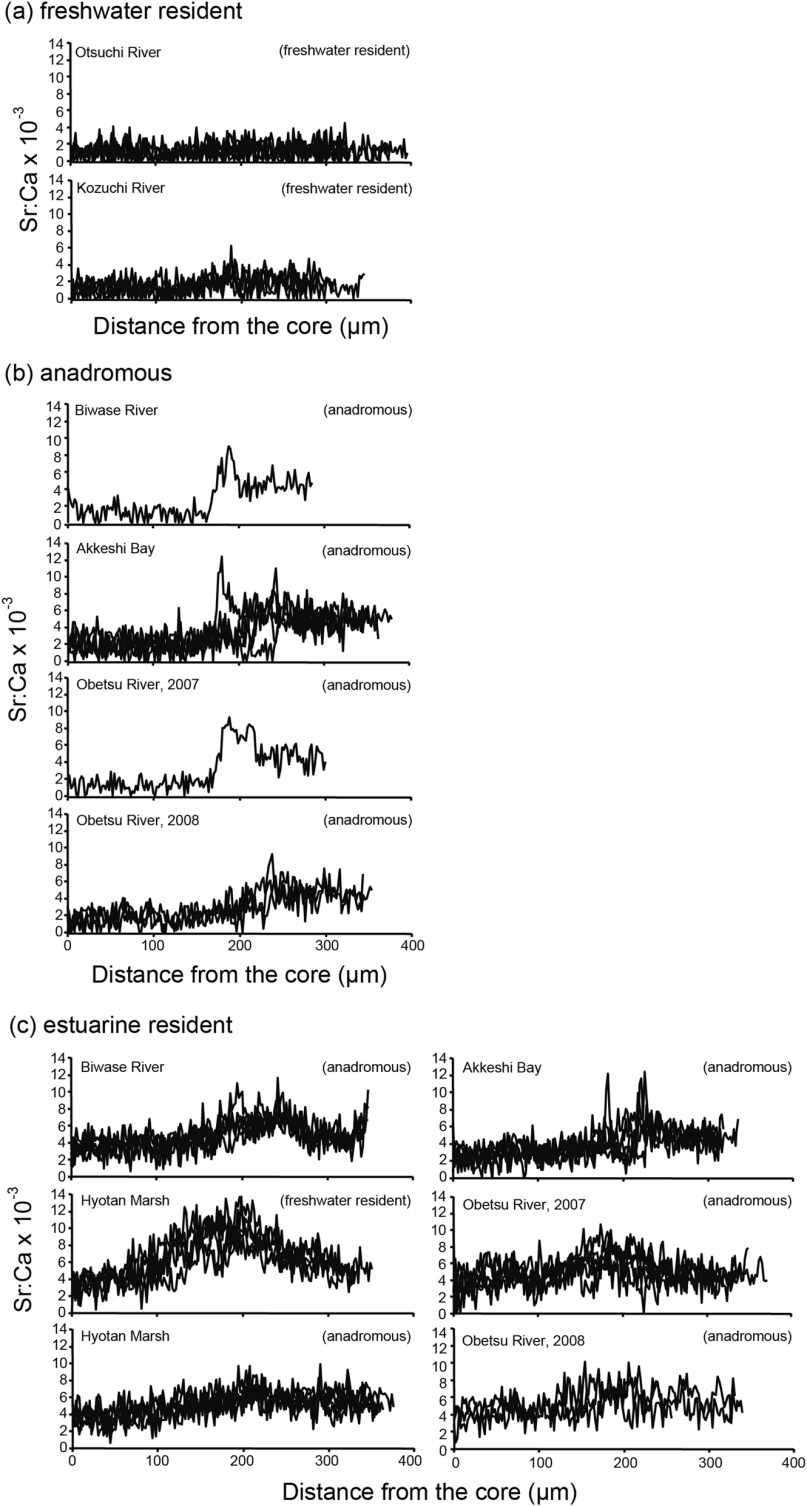
Migratory history of threespine stickleback *G. aculeatus* as indicated by the temporal pattern of the Sr:Ca ratios in their otoliths. Plots of otolith Sr:Ca ratios along a transect line from the core to the edge of the otolith were divided into freshwater resident (**a**), anadromous (**b**) and estuarine resident (**c**). Life history type, as determined by morphological characteristic, is indicated in the upper right corner in each plot.

**Figure 3:**
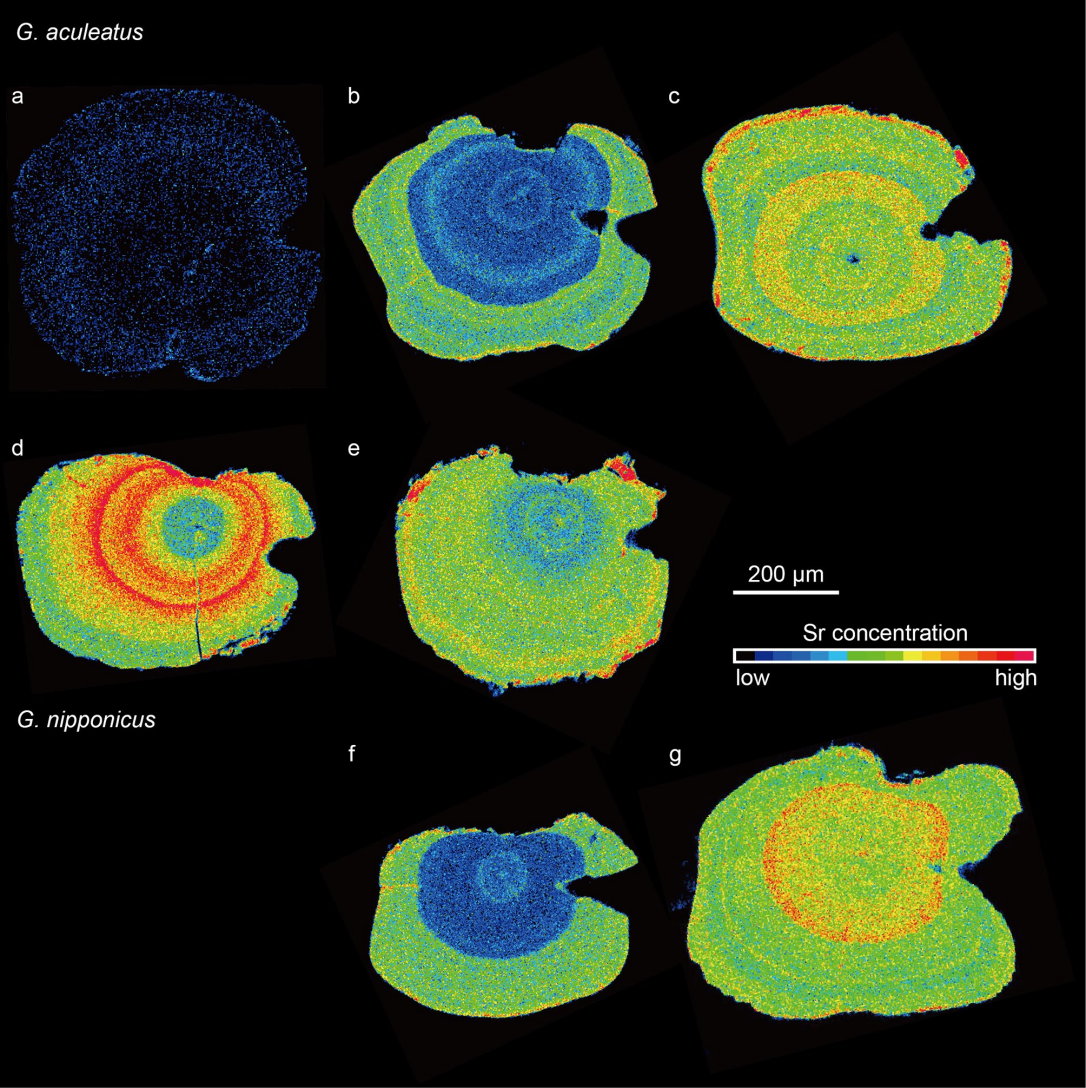
Two-dimensional images made using X-ray electron microprobe analysis of the Sr concentrations in the otoliths of threespine sticklebacks *Gasterosteus aculeatus* and *G. nipponicus* in northern Japan. Freshwater resident (**a**) migration pattern of a *G. aculeatus* morphologically freshwater resident specimen, anadromous (**b**) and estuarine resident (**c**) migration patterns in *G. aculeatus* of morphologically anadromous specimens, estuarine resident migration patterns in *G. aculeatus* of morphologically freshwater resident (**d**) and anadromous (**e**) specimens collected in Hyotan Marsh and anadromous (f) and estuarine resident (**g**) migration patterns in *G. nipponicus* of morphologically anadromous specimens. Sr concentrations are represented by 16 colours from red (highest) to yellow to green to blue (lowest).

There were three migratory types, i.e., freshwater resident, anadromous and estuarine resident, in *Gasterosteus aculeatus* (Fig. 2). The freshwater residents showed consistently low Sr:Ca ratios along a line transect, averaging 1.39 × 10^–3^ ± 0.22 × 10^–3^ (± SD) (range: 1.14–1.80 × 10^–3^) (Fig. 2a), characterized by a bluish colour (low Sr concentration) from the otolith core to the edge in the otolith X-ray intensity map (Fig. 3a). We found 35 of 144 (24.3%) specimens were freshwater residents, suggesting continuous residence in freshwater environments after hatching, although the habitats (Otsuchi and Kozuchi rivers) directly flow to the sea (Otsuchi Bay) and the fish can migrate downstream to the sea (Fig. 1). The anadromous showed a low Sr:Ca ratio phase from the core to the point approximately 170–200 µm (phase L), averaging 1.93 × 10^–3^ ± 0.41 × 10^–3^ (± SD) (range 1.26–2.38 × 10^–3^), which corresponds to freshwater life period. Thereafter, the ratios increased sharply, averaging 4.96 × 10^–3^ ± 0.51 × 10^–3^ (± SD) (range 4.13–6.15 × 10^–3^) and were maintained at higher levels until the outermost regions corresponded to a brackish water or seawater life period (phase H, Fig. 2b). Significant differences occurred in the Sr:Ca ratios between phase L and phase H in the 17 anadromous specimens (Mann Whitney-*U* test, df = 84–180, U = 41–1,236, *p* < 0.0001). The anadromous showed a wide space of bluish colour (low Sr) radiating from the centre, which was surrounded by concentric rings having higher Sr concentrations in the otolith X-ray intensity map (Fig. 3b), which corresponded to the otolith Sr:Ca ratios along a line transect. We found 11.8% (17 of 144) sticklebacks showed a typical anadromous migration history, having a clear transition point (TP) from phase L to phase H in a line transect (Fig. 2b). The estuarine residents, which did not have a clear TP along a life history transect, showed relatively high Sr:Ca ratios consistently from the core to the edge, averaging 4.94 × 10^–3^ ± 0.53 × 10^–3^ (range 4.55–5.93 × 10^–3^) (Fig. 2c). The estuarine residents were characterized by reddish and greenish colours (higher Sr concentration) from the otolith core to the edge in the otolith X-ray intensity map (Fig. 3c). We found 92 of 144 (63.9%) sticklebacks showed consistently high Sr:Ca ratios along a line transect, although these specimens were classified either as anadromous or freshwater residents based on morphology. Freshwater resident and anadromous sticklebacks as inferred by morphology and the habitat environments occurred sympatrically in Hyotan Marsh, and the TL in the freshwater residents (mean ± SD; 70.1 ± 3.7) was significantly smaller than that of the anadromous (87.5 ± 4.7) (Mann Whitney-*U* test, df = 13, U = 11, *p* < 0.0001), while their life histories were both estuarine residents (Fig. 2c, Fig. 3d,e, Table 1). Interestingly, otolith Sr:Ca ratios from the Hyotan Marsh of the morphologically freshwater resident type showed a slight increase around between 150 and 250 μm from the core, and then it decreased again to the otolith edge (Fig. 2c, Fig. 3d). The mean TLs of freshwater residents, anadromous and estuarine residents in all *G. aculeatus* specimens as determined by otolith Sr:Ca ratios were 68.6 ± 5.8 mm (mean ± SD), 88.9 ± 6.2 mm and 87.6 ± 8.7 mm, respectively. There were significant differences in mean TLs among the three migration types (Kruskal–Wallis test, n = 143, H = 65.568, *p* < 0.0001). Significant differences were found among freshwater residents and anadromous and estuarine residents (Mann Whitney-*U* test, df = 27–91, U = 2–164, *p* < 0.0001) while no significant difference was found between anadromous and estuarine resident (Mann Whitney-*U* test, df = 26, U = 727.5, *p* > 0.05).

**Table 1:** Threespine sticklebacks, *Gasterosteus aculeatus* and *G. nipponicus*, used in the present study.

Sampling location	Sampling date	Number of Specimens examined	Total length (mm)		Life history style estimated from morphology	Water environment/salinity	Migration pattern estimated from otolith Sr:Ca ratios	Number of specimens	Otolith Sr:Ca ratios	Total length (mm)
			Mean ± SD	Range					Mean ± SD	Mean ± SD
***G. aculeatus***										
Biwase River	June 2007	23	93.3 ± 4.2	83.7–99.8	Anadromous	20	Anadromous	1	2.7	91.2
							Estuarine resident	22	4.9 ± 1.6	93.4 ± 4.3
Hyotan Marsh	June 2007	10	70.1 ± 3.7	62.8–76.8	Freshwater resident	16–31	Estuarine resident	10	6.0 ± 2.4	70.1 ± 3.7
		6	87.5 ± 4.7	85.3–90.6	Anadromous		Estuarine resident	6	5.1 ± 1.5	87.5 ± 4.7
Obetsu River	June 2007	10	92.4 ± 4.2	87.3–99.5	Anadromous	Intertidal	Anadromous	1	3.1	87.4
							Estuarine resident	9	4.6 ± 1.7	92.9 ± 4.1
	June 2008	30	93.2 ± 4.1	79.3–99.8	Anadromous		Anadromous	8	3.5 ± 2.1	91.8 ± 6.1
							Estuarine resident	22	4.5 ± 1.7	93.7 ± 3.1
Akkeshi Bay	June 2004	30	82.2 ± 4.1	74.8–90.4	Anadromous	Intertidal	Anadromous	7	3.2 ± 1.9	83.6 ± 3.9
							Estuarine resident	23	4.5 ± 1.7	81.5 ± 4.2
Otsuchi River	May 2007	20	70.7 ± 4.5	64.6–78.3	Freshwater resident	0	Freshwater resident	20	1.2 ± 0.9	70.7 ± 4.5
Kozuchi River	May 2009	15	65.7 ± 6.4	51.6–76.6	Freshwater resident	0	Freshwater resident	15	1.6 ± 1.1	65.7 ± 6.4
***G. nipponicus***										
Cape Soya	June 2009	26	75.6 ± 4.6	65.5–84.5	Anadromous	Intertidal	Anadromous	1	3.8 ± 1.9	65.5
							Estuarine resident	25	4.6 ± 1.5	76.0 ± 4.2
Toyokanbetsu River	June 2009	30	79.0 ± 4.4	67.7–88.6	Anadromous	Intertidal	Estuarine resident	30	5.2 ± 1.7	79.0 ± 4.4
Lake Saroma	June 2009	5	76.4 ± 6.3	68.2–83.1	Anadromous	Intertidal	Estuarine resident	5	4.6 ± 1.4	76.4 ± 6.3
Biwase River	June 2007	30	67.9 ± 11.7	52.2–85.4	Anadromous	20	Estuarine resident	30	5.2 ± 1.6	67.9 ± 11.7
Biwase River	June 2007	30	75.3 ± 4.3	68.0–88.7	Anadromous	Intertidal	Anadromous	1	4.0	68.0
Tidal pool							Estuarine resident	29	5.0 ± 1.5	75.6 ± 4.2
Hyotan Marsh	June 2007	6	79.0 ± 3.3	76.9–81.7	Anadromous	16–31	Estuarine resident	6	5.1 ± 1.5	79.0 ± 3.3
Shiomi River	June 2007	22	67.0 ± 11.8	51.1–84.6	Anadromous	1–28	Estuarine resident	22	5.1 ± 1.7	67.0 ± 11.8
Obetsu River	June 2007	30	77.1 ± 3.6	67.2–84.7	Anadromous	Intertidal	Estuarine resident	30	5.2 ± 1.6	77.1 ± 3.6
Numajiri River	June 2007	30	67.5 ± 10.7	51.6–82.7	Anadromous	3–4	Anadromous	3	3.4 ± 2.2	76.2 ± 4.0
							Estuarine resident	27	4.9 ± 1.6	66.5 ± 10.8
	June 2008	30	76.7 ± 4.2	65.1–84.1	Anadromous		Anadromous	1	3.8	74.9
							Estuarine resident	29	5.6 ± 1.8	76.8 ± 4.3
Lake Takkobu	June 2007	30	75.9 ± 4.1	69.0–82.6	Anadromous	0	Anadromous	28	3.3 ± 2.1	75.9 ± 3.9
							Estuarine resident	2	4.9 ± 1.3	75.4 ± 8.7
Lake Ogawara	May 2004	60	79.9 ± 3.7	72.4–89.9	Anadromous	Intertidal	Anadromous	1	3.6	88.5
							Estuarine resident	59	5.1 ± 1.5	79.8 ± 3.6
	April 2005	60	77.7 ± 4.3	77.1–84.3	Anadromous		Anadromous	13	3.6 ± 2.0	77.3 ± 3.9
							Estuarine resident	47	5.3 ± 1.6	77.0 ± 4.4
	May 2006	60	77.7 ± 4.7	69.2–88.8	Anadromous		Anadromous	7	3.3 ± 1.9	83.1 ± 5.9
							Estuarine resident	53	5.1 ± 1.5	77.0 ± 4.1
Miyako Bay	June 2004	3	69.6 ± 13.1	55.4–81.2	Anadromous	Intertidal	Estuarine resident	3	6.5 ± 2.1	69.6 ± 13.1
	May 2007	10	75.6 ± 4.3	69.9–81.6	Anadromous		Anadromous	1	3.3	78.6
							Estuarine resident	9	5.4 ± 1.6	75.3 ± 4.5
Funakoshi Bay	June 2006	2		75.6–80.7	Anadromous	Intertidal	Estuarine resident	2	6.9 ± 1.9	75.6–80.7
Otsuchi Bay	June 2004	1		77.7	Anadromous	Intertidal	Estuarine resident	1	8.1	77.7
	June 2006	2		70.7–74.2	Anadromous		Estuarine resident	2	7.3 ± 2.3	70.7–74.2
Okirai Bay	September 2007	10	60.1 ± 1.8	57.2–62.8	Anadromous	Intertidal	Estuarine resident	10	5.6 ± 1.7	60.1 ± 1.8

**Figure 4:**
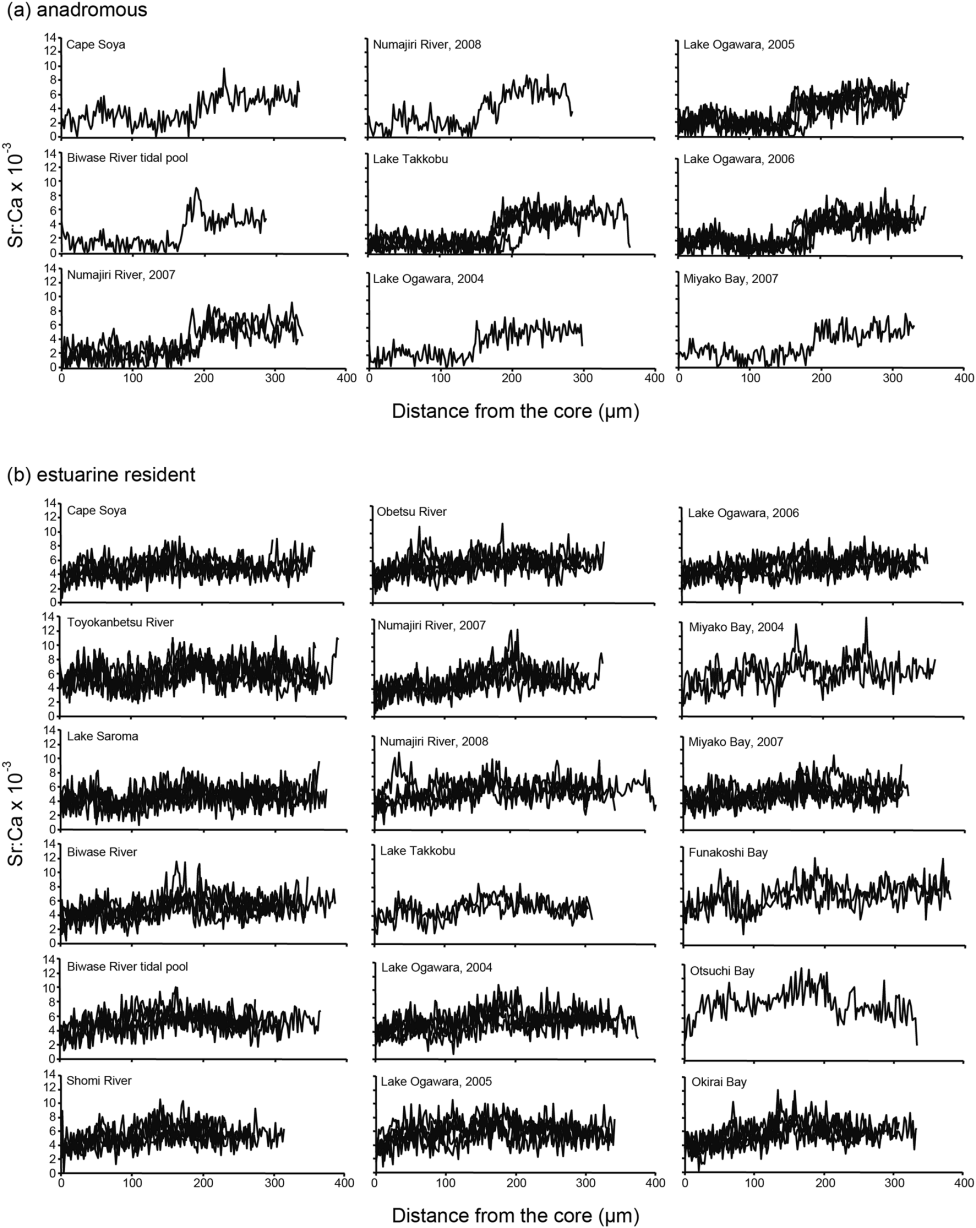
Migratory history of threespine stickleback *Gasterosteus nipponicus* as indicated by the temporal pattern of the Sr:Ca ratios in their otoliths. Plots of otolith Sr:Ca ratios along a transect line from the core to the edge of the otolith were divided into anadromous (**a**) and estuarine resident (**b**).

In *G. nipponicus*, there were two migratory types, i.e. anadromous and estuarine residents, as determined from otolith Sr:Ca ratios along a line transect and otolith X-ray intensity maps of the otolith Sr content (Figs. 3, 4). Otolith micochemical signatures could identify anadromous among a total of 56 in 477 specimens, and all of the rest of sticklebacks (421 specimens) were estuarine residents (Table 1). We found 56 of 477 (11.7%) sticklebacks had a TP at approximately 150–190 µm along a line transect and showed typical anadromous (Fig. 4a). The mean Sr:Ca ratios in phase L and phase H averaged 1.88 × 10^–3^ ± 0.32 × 10^–3^ (± SD) (range: 0.97–2.23 × 10^–3^) and 5.24 × 10^–3^ ± 0.42 × 10^–3^ (range: 4.21–6.43 × 10^–3^), respectively, and those phases were significantly different (Mann Whitney-*U* test, df = 18–185, U = 0–715, *p* < 0.0001). Two-dimensional images of the Sr concentration in the otoliths showed a wide space of bluish colour (low Sr) radiating from the centre, which was surrounded by higher Sr concentrations (Fig. 3f). In Lake Takkobu, a freshwater lake, 28 of 30 sticklebacks showed the anadromous, and a slight decrease in otolith Sr:Ca ratio was found around the edge in 16 of the 28 sticklebacks. Ten of the 16 fishes had an otolith Sr:Ca ratio less than 2.5 × 10^–3^ (mean ± SD: 1.85 × 10^–3^ ± 0.56 × 10^–3^, range: 0.75–2.41 × 10^–3^). The low Sr:Ca ratio in the otolith edge corresponds to a short freshwater period during spawning migration in *G. nipponicus*. The estuarine resident was the dominant migratory pattern, constituting 88.3% (421 of 477) of the sticklebacks. The estuarine residents showed consistently high Sr:Ca ratios from the core to the edge (Fig. 4b), averaging 5.58 × 10^–3^ ± 0.93 × 10^–3^ (range: 4.60–8.09 × 10^–3^), characterized by a higher Sr concentration throughout the whole otolith in the otolith X-ray intensity map (Fig. 3g). Although *G. nipponicus* is thought to be only an anadromous based on its morphology and habitat environments, most of the sticklebacks showed an estuarine resident without any signs of freshwater life (Fig. 4b), similar to *G. aculeatus*.

There were no significant differences in the mean Sr:Ca ratios of estuarine residents between *G. aculeatus* and *G. nipponicus* (Mann Whitney-*U* test, df = 15, U = 26, *p* > 0.05). No significant differences in the mean Sr:Ca ratios of phase L and phase H in anadromous were also found between species (Mann Whitney-*U* test, df = 21–23, U = 279–457, *p* > 0.05). These results suggested there is no significant interspecific variation in otolith Sr:Ca ratio.

### Habitat use

The mean otolith Sr:Ca ratios from the core to the edge in freshwater resident, anadromous and estuarine resident were 1.39 × 10^–3^ ± 0.22 × 10^–3^ (mean ± SD), 3.39 × 10^–3^ ± 0.45 × 10^–3^ and 5.13 × 10^–3^ ± 0.68 × 10^–3^, respectively. There were significant differences in the mean otolith Sr:Ca ratios among the three migration types (Kruskal–Wallis test, n = 622, H = 261.013, *p* < 0.005) and significant differences were found in all combinations (Mann Whitney-*U* test, df = 90–127, U = 0–556, *p* < 0.0001). The mean otolith Sr:Ca ratios can be used as a new indicator to differentiate habitat use in threespine sticklebacks because morphological analyses do not necessarily reflect their life history patterns (Table 1).

**Figure 5:**
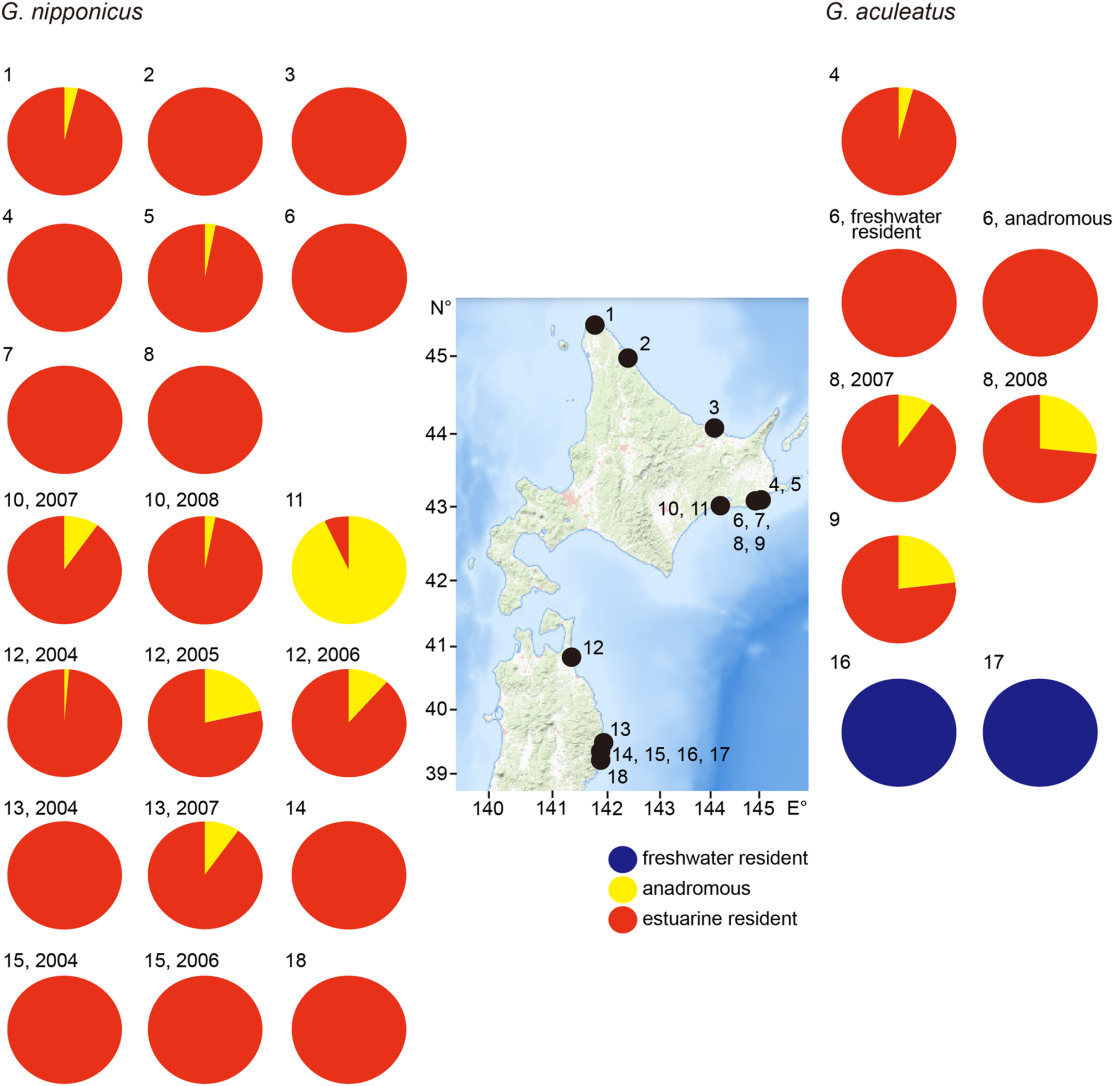
Habitat use of threespine sticklebacks, *Gasterosteus aculeatus* and *G. nipponicus,* in northern Japan. Composition of habitat uses, i.e., freshwater resident, anadromous and estuarine resident, of *G. aculeatus* (right) and *G. nipponicus* (left) in each location. Blue: freshwater resident, yellow: anadromous, red: estuarine resident. Numbers with the black dot on the map of northern Japan correspond to each sampling site. 1, Cape Soya, 2, Toyokanbetsu River, 3, Lake Saroma, 4, Biwase River, 5, Biwase River tidal pool, 6, Hyotan Marsh, 7, Shiomi River, 8, Obetsu River, 9, Akkeshi Bay, 10, Numajiri River, 11, Lake Takkobu, 12, Lake Ogawara, 13, Miyako Bay, 14, Funakoshi Bay, 15, Otsuchi Bay, 16, Otsuchi River, 17, Kozuchi River, 18, Okirai Bay. The base map was downloaded from the USGS National Map Viewer (open access) at https://viewer.nationalmap.gov/viewer/.

*Gasterosteus aculeatus* in the Otsuchi and Kozuchi rivers in complete freshwater environments above the intertidal areas were all freshwater residents (100%) (Fig. 5). Morphologically anadromous specimens of *G. aculeatus* were mostly estuarine residents, averaging 89.3 ± 11.8% (± SD) and ranging from 73 to 100% at each site (Fig. 5), while the remaining fishes (mean ± SD; 10.7 ± 11.8%, range 0% to 27%) were typical anadromous (Fig. 5). Interestingly, in Hyotan Marsh, otolith Sr:Ca ratios in morphologically freshwater resident fishes (mean ± SD; 6.0 × 10^–3^ ± 2.4 × 10^–3^) were significantly higher than those of morphologically anadromous fishes (5.1 × 10^–3^ ± 1.5 × 10^–3^) (Mann Whitney-*U* test, df = 11, U = 4, *p* < 0.01), although both sticklebacks were identified as estuarine residents by means of the otolith microchemical signatures.

In *G. nipponicus*, habitat use in 14 of 15 sites in morphologically anadromous was mostly estuarine resident, averaging 95.6 ± 6.4% (± SD) and ranging from 78 to 100% (Fig. 5), while only a few remaining individuals (mean ± SD; 4.4 ± 6.4%, range 0% to 22%) were actually anadromous (Fig. 5). Meanwhile, 93% of sticklebacks from the Lake Takkobu were typical anadromous, while 7% were estuarine residents.

The interannual variability of the habitat use was examined in Ohbetsu River (2 years) for *G. aculeatus* and Numajiri River (2 years) and Lake Ogawara (3 years) for *G. nipponicus* in the same period (Table 1, Fig. 5). The deviations of interannual variations were 5 to 12% in each site, and the composition of each habitat use was constant for several years. Furthermore, the occurrence of the “estuarine resident” novel life history found previously^26,27^ was temporally and spatially a general phenomenon in these threespine sticklebacks as their dominant migration pattern.

## Discussion

The validity of otolith Sr concentration and Sr:Ca ratios to reconstruct the migratory history and habitat use in habitats including freshwater, brackish water, and seawater was first demonstrated in threespine sticklebacks in the present study. Otolith Sr and Sr:Ca ratios were positively correlated with salinity. The intercept of the linear regression between the otolith Sr:Ca ratio and salinity was 2.1 × 10^–3^ at zero salinity. This result suggests that an otolith Sr:Ca ratio of 2.5 × 10^–3^ can discriminate between a freshwater life history and a saltwater one in threespine sticklebacks. This study found that threespine sticklebacks had three migratory types including freshwater residents, anadromous and estuarine residents based on their otolith microchemical signatures. Anadromous stickleback had a higher Sr:Ca ratio phase (phase H), and the ratio increased abruptly after a lower Sr:Ca ratio phase (phase H) with a clear transition point (TP) along a line transect. Estuarine resident sticklebacks had consistently high Sr:Ca ratios from the core to the edge along a line transect. The mean high Sr:Ca ratios found in wild sticklebacks were more than 4.5 × 10^–3^ and were slightly higher than the sticklebacks reared in the full seawater (4.1 × 10^–3^). Such a difference in otolith Sr:Ca ratios between reared and wild fishes has also been found in salmonids^39,40^. The reason for this discrepancy is unclear, but it may be related to population-specific differences in Sr incorporation at higher salinity or to ontogenic-related changes in osmoregulatory capacity. The somatic growth in sticklebacks reared in full salinity (30 psu) was significantly lower than those reared in 20 psu. The experimental sticklebacks reared in full salinity would not be in optimal condition and may have had different osmotic adaptations in relation to incorporation mechanisms of otolith Sr than those in wild sticklebacks. Otherwise, it would not be plausible to rear juvenile sticklebacks to seawater just after hatching. Although Sr has been generally considered as a powerful tool to differentiate between freshwater and marine/brackish water environments, species-specific variations, environmentally mediated physiological variations and multiple environmental factors can influence otolith Sr incorporation^41–45^. Further studies on the ontogenic effects and influences of osmoregulation and physiological changes together with other factors such as water temperature and diet on the incorporation of Sr and Ca in otoliths are needed to elucidate a plausible mechanism. In any case, based on a positive linear relationship between salinity and otolith Sr:Ca ratios, the migratory history can be reconstructed from otolith Sr:Ca ratios in threespine sticklebacks.

**Figure 6:**
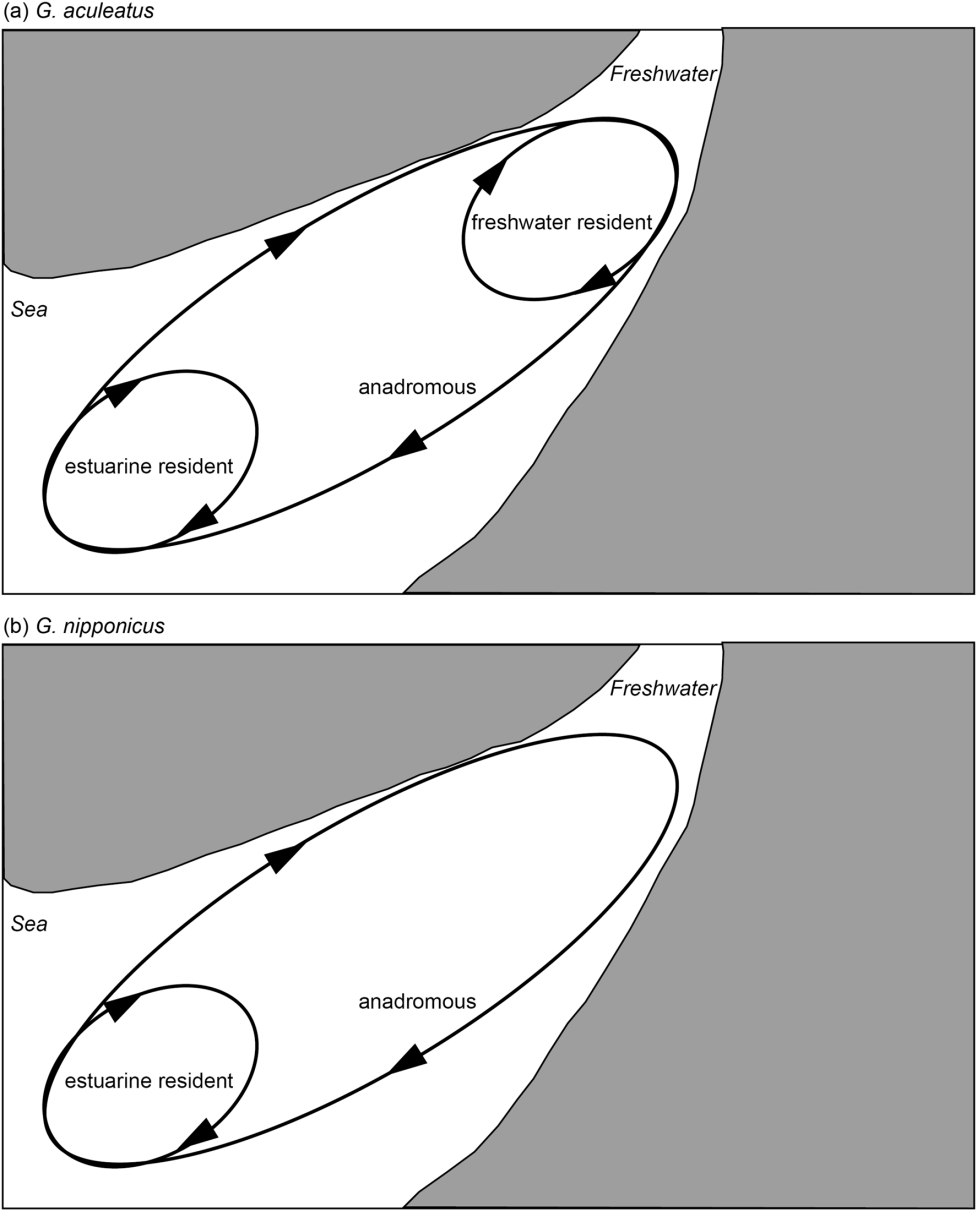
Migratory histories of threespine sticklebacks, *Gasterosteus aculeatus* and *G. nipponicus*, as revealed by otolith microchemical signatures. *G. aculeatus* comprises freshwater residents, anadromous and estuarine residents (**a**) and *G. nipponicus* comprises anadromous and estuarine residents (**b**).

Based on the life history transect and mean value of the otolith Sr:Ca ratio, migratory history and habitat use of threespine sticklebacks, *Gasterosteus aculeatus* and *G. nipponicus* could be divided into three types, i.e., freshwater resident, anadromous and estuarine residents. Differences in migratory patterns were found between *G. aculeatus* and *G. nipponicus*, i.e., *G. aculeatus* comprises freshwater resident, anadromous and estuarine resident (Fig. 6a), while *G. nipponicus* comprises anadromous and estuarine resident (Fig. 6b). The estuarine resident was defined as a stickleback that had a continuous high Sr:Ca ratio from the core to the edge of otolith without a transition point in the otolith Sr:Ca ratios from a low phase to a high phase^27^. The estuarine resident was the major migratory pattern in both species, constituting approximately 90% of the morphologically anadromous sticklebacks while the actual anadromous were only 10%. The estuarine residents occurred in almost all (16 of 18) sites, including a freshwater environment (Lake Takkobu) with little interannual variation. The occurrence of the estuarine resident showed only limited spatial and temporal variations in northern Japan. These results suggest that the estuarine resident was the general migratory pattern in intertidal habitats in threespine sticklebacks. At the moment, threespine sticklebacks are differentiated based on their morphologic characteristics and habitat environment^7,24,25,30^. However, the present results showed discrepancies in migratory history and habitat use between morphological and habitat indications and otolith Sr:Ca signatures (Table 1). Especially, morphologically freshwater residents of *G. aculeatus* in the Hyotan Marsh were estuarine residents as inferred from otolith Sr:Ca ratios. The mean otolith Sr:Ca ratios in morphologically freshwater resident *G. aculeatus* (6.0 × 10^–3^) were significantly higher than those of morphologically anadromous *G. aculeatus* (5.1 × 10^–3^) (but both types were estuarine residents by otolith microchemistry). The freshwater resident *G. aculeatus* lived in higher saline environment than the anadromous *G. aculeatus* in the marsh. The freshwater resident *G. aculeatus* might shift habitats (salinity environments) during their lives due to the otolith Sr:Ca ratios were slightly increased around between 150 and 250 μm from the core, and then it decreased again to the otolith edge (Figs. 2c, 3d). Significant differences in growth (TL) between those anadromous (large-sized: 87.5 mm TL in average) and freshwater residents (small-sized: 70.1 mm TL) of *G. aculeatus* were found in the marsh. The habitat uses (mean otolith Sr:Ca ratios) and the migratory history patterns (line transects) were different between these two sticklebacks (Fig. 2c). The large-sized sticklebacks consistently lived in the same saline environment while the smaller-sized fishes shifted their habitats. The small-sized fishes may have migrated to higher-salinity downstream areas of a bay (Akkeshi Bay) and tidal tributaries and then migrated back to the marsh for spawning. Differences in migratory history and habitat use might explain the two different types of growth. Almost all morphologically anadromous sticklebacks of both *G. aculeatus* and *G. nipponicus* actually showed an estuarine resident (Table 1, Fig. 5). The otolith microchemistry can trace the individual life history from birth to death, while classical morphological characteristic and habitat environment observations do not necessarily reflect the individual’s life history. Therefore, the terms “freshwater” and “anadromous” based on the morphological characteristics and habitat environments that have widely been used, are not accurate to differentiate the life history and migration patterns of threespine sticklebacks.

Approximately 90% of *G. aculeatus* and *G. nipponicus* in intertidal areas spent their entire lives in brackish water and/or seawater environments, although these sticklebacks were believed to spawn and spend their early life in a freshwater environment as part of an anadromous till now. A few studies found that *G. aculeatus* spawned in coastal regions such as tidal pools, salt marshes and estuaries^1,6,46,47^. *G. aculeatus* was found to spawn in seawater environments such as tidal pools in Canada^48^. Sticklebacks, including nearly mature individuals, have been collected up to 500–800 km offshore, suggesting the fishes could reach full spawning condition in the sea^49,50^. *G. nipponicus* was also found to spawn in seawater tidal pools of western Hokkaido Island, Japan, in our previous study^35^. All tidal pools are high salinity environments, ranging from 33 to 35 psu. Several males built their nests in the pools every year, and the females also collected in the tidal pools. The otolith Sr:Ca ratios collected from the seawater tidal pools were consistently higher from the core to the edge in both adult (62.6–79.1 mm in TL) and juvenile (22.1–25.4 mm in TL) *G. nipponicus* specimens^35^, averaging 5.6 × 10^–3^ to 5.8 × 10^–3^. Therefore, sticklebacks in tidal pools can survive in full seawater environments during the maturation period, as eggs and newly hatched juveniles. The present and previous studies suggest that most sticklebacks live in intertidal areas such as estuaries, salt marshes and tidal pools, and could be estuarine residents that complete their entire lives in seawater and/or brackish water environments without any freshwater life. Several scenarios of life history strategies, including reproduction, growth and survival, could account for the dominant occurrence of estuarine residents rather than anadromous and freshwater residents, in *G. aculeatus* and *G. nipponicus*. First, spawning migration from marine to freshwater habitats in anadromous sticklebacks may have an energetic expenditure due to the need for further swimming, and the other cost may be a physiological one accompanied by osmotic and ionic regulation between environments with different salinity levels. Second, the estuarine resident may benefit in growth and survival from continuously residing in more productive coastal habitats than anadromous and freshwater fishes. The primary production in marine habitats is higher than that in freshwater habitats at the higher latitudes^51^ where threespine sticklebacks are distributed. The estuarine residents would experience enhance larval and juvenile feeding and thus grow and survive. Indeed, the growth of estuarine resident *G. aculeatus* fishes was significantly greater than that of freshwater resident individuals.

There occurred two migration types, anadromous and estuarine resident, in a freshwater lake (Lake Takkobu) (Table 1, Figs. 4, 5) in *G. nipponicus*. Those migration patterns were found in our previous study conducted in a different year (May 2001)^26^. In the present study, the majority (93%) of anadromous sticklebacks were only found in the lake, and the remaining sticklebacks (7%) were estuarine residents (Fig. 5). We found 36% of the sticklebacks in the lake had a clearly low otolith Sr:Ca ratio, less than 2.5 × 10^–3^, with 57% fishes having a decreased ratio around the edge. This suggests that these sticklebacks have recently immigrated into the lake for spawning. Anadromous sticklebacks migrate into freshwater areas from the sea to spawn in the spring and early summer^1^, and the sticklebacks used in this study were collected during the spawning season (June). Accordingly, a low Sr:Ca ratio would not be incorporated into the edge of otolith in some sticklebacks. Interestingly, both anadromous and estuarine resident sticklebacks were sympatrically found in the freshwater lake. In the wild, anadromous sticklebacks were found to school strongly while freshwater resident sticklebacks exhibit reduced schooling^3,19^. The estuarine resident sticklebacks hatched and lived in the downstream brackish water and/or seawater environments. However, those sticklebacks might migrate upstream to the lake together with anadromous sticklebacks for spawning. The progeny of the estuarine resident *G. nipponicus* would eventually become an anadromous instead of estuarine residents in the lake. This suggests the hypothesis that stickleback migration may be rather opportunistic and determined by ecological plasticity and social behaviour more so than genetic traits. Therefore, sticklebacks can flexibly live in various environments such as freshwater, brackish water or seawater.

In our present and previous studies^26,27^, we defined “estuarine residents” that complete their entire lives in seawater and/or brackish water environments without any freshwater life. Except for the European and eastern North American clade, some marine populations spawned in brackish water and/or freshwater^31,52–55^. The other marine populations in immature sticklebacks were collected from open ocean and/or coastal waters^46,47^. The results suggest that *G. aculeatus* spawning in full seawater habitats is distributed in only eastern North American region^56^. We did not define “marine residents” in the present study due to the all “estuarine residents” were collected in intertidal areas and we could not determine whether they completed full seawater environments throughout their lives or not. Furthermore, we could not properly discriminate between seawater and brackish water habitats by means of otolith Sr:Ca ratios. Further comparative ecological and physiological studies of *G. aculeatus* and *G. nipponicus* among a number of breeding populations from different salinity environments are needed to define migration types to understand deeply the diversity of life history in threespine sticklebacks.

Fishes exhibit a high level of diversity in migration patterns, and there are many arguments about the advantages and disadvantages of fish migration^57–60^. Advantages include optimal foraging, predator avoidance, and enhancing reproductive fitness, while disadvantages include energetic expenditure, osmoregulatory stress, and increased predation risk^57–60^. Optimal trade-offs between benefits and costs differ between habitat environments and can stimulate the occurrence of diverse migratory behaviours. Many of the Gasterosteiformes are marine species and hence the marine spawning habits in threespine sticklebacks is probably a conservative trait. There is no occurrence of freshwater resident life history in *G. nipponicus*, and this would also support the marine origin of threespine sticklebacks. Immigration into freshwater habitats for spawning in the anadromous life pattern may have energy and osmoregulatory costs. Dominant occurrence of estuarine residents in *G. aculeatus* and *G. nipponicus*, therefore, would have resulted in the optimal trade-offs in those species. In *G. aculeatus*, however, freshwater residents were found in many rivers, streams and ponds in the previous^7,26,27^ and present (Otsuchi and Kozuchi rivers) studies, although freshwater residents never occurred among *G. nipponicus*. However, the population size of the freshwater resident in *G. aculeatus* may be much smaller than those of the anadromous and estuarine residents based on the distribution range of *G. aculeatus* in the Japanese archipelago^24,61,62^. Freshwater resident sticklebacks are believed to be derived from anadromous sticklebacks^4^. However, the present results suggest that anadromous sticklebacks may have evolved from estuarine residents, because most of the morphologically anadromous sticklebacks were actually estuarine residents, and the estuarine resident migration type has newly been discovered together with their relative composition. The present study leads to a possible scenario of the evolution of migration in threespine sticklebacks (Fig. 6). Some estuarine resident sticklebacks gradually migrated and expanded their growth habitat into freshwater environments and regularly migrated between sea (brackish) water and freshwater habitats as an anadromous in *G. aculeatus* and *G. nipponicus*, and thereafter, some anadromous sticklebacks never migrated back to estuarine/marine habitats and settled permanently in freshwater environments as the freshwater residents of *G. aculeatus* (Fig. 6).

In threespine sticklebacks (*G. aculeatus* and *G. nipponicus*) examined from various environments in northern Japan, the present study first demonstrated that approximately 90% of anadromous sticklebacks had estuarine resident migration pattern. The dominant occurrence of the estuarine resident was temporally and spatially consistent with their general migration ecology. The estuarine resident is thought to be the ancestral migrations of *G. aculeatus* and *G. nipponicus*, which thereafter gradually immigrated into freshwater habitats and settled in the anadromous form in both species and finally became the freshwater resident *G. aculeatus*. Threespine sticklebacks are widely distributed across the northern hemisphere. However little is known about their migration ecology across the whole distribution rage. Further studies, such as genetic and ecological studies in combination with otolith analyses need to be carried out to understand the mechanisms of migration and habitat selection for a wide range of areas and the actual divergence mechanism for the life histories of the species.

## Methods

### Relationship of otolith Sr:Ca ratios and salinity

The newly hatched *Gasterosteus aculeatus* juveniles were used in the experiment, and their parents were collected from the Hyotan Marsh of the Shiomi River basin in June 2008. To examine the relationship between the Sr and Ca concentrations in the otoliths and various ambient salinity conditions, 40 juveniles divided equally into four tanks (10 juveniles in each tank) were each reared for 30 days in either 0 psu, 10 psu, 20 psu, or 30 psu and half of the water for all tanks was changed every 2 weeks during June and July 2008. In the experiment, the tanks were all placed in one room to make sure the experimental tanks were in the same environmental condition. The fishes were fed once daily, and the unconsumed food was removed. The water temperature was not controlled in the experiment; however, the room temperature was 20 ± 1 °C, and it affected the temperature of the aquariums. The rearing water was filtered using a 0.45-µm filter and diluted with Milli-Q water before the Sr and Ca concentrations were measured by an inductively coupled plasma mass spectrometer (ICP-MS) (Agilent-7500cs, Agilent Technology, Tokyo, Japan). The isotope intensities of ^88^Sr and ^44^Ca were calibrated to Sr and Ca concentrations, respectively, using those of standard waters with certified abundances after the subtraction of blanks. TL of each fish was measured after the experiment. In addition, ten wild *G. aculeatus* juveniles (range 18.6–26.0 mm, mean ± SD; 22.2 ± 2.1 mm in TL) were collected from an artificial pond that was a completely isolated environment on the campus of Kitasato University in Iwate, Japan and were used to understand the freshwater benchmark of otolith Sr:Ca ratios in threespine sticklebacks reared under a natural environment. Our protocols followed the ethical guidelines for the use of animals of Universiti Brunei Darussalam (UBD) and were approved by the animal ethics committee at UBD throughout this research.

### Fish collection

A total of 621 threespine sticklebacks consisted of 144 *Gasterosteus aculeatus* and 477 *G. nipponicus* were collected by dip, casting and seine nets in environments of various salinities (0–31 psu; freshwater to seawater) such as rivers, lakes, marshes, estuaries, tidal ponds, and coastal waters at a total of 18 sites (6 sites in *G. aculeatus* and 15 sites in *G. nipponicus*) in northern Japan from 2004 to 2009 (Fig. 1, Table 1). *G. aculeatus* specimens from 4 of 6 sites were collected from intertidal zones (Table 1). *G. aculeatus* specimens in freshwater habitats were collected from the Otsuchi and Kozuchi rivers in northeastern Japan. These sampling sites were above the intertidal area and were not influenced by the rising tide, although these rivers flow into Otsuchi Bay (Fig. 1). *G. nipponicus* specimens from 14 of 15 sites except for Lake Takkobu, in eastern Hokkaido Island, Japan, were collected from intertidal zones (brackish water or seawater habitats) that were influenced by the rising tide (Table 1). Lake Takkobu, an inland sea-lake, is connected to the sea by an intermittent small stream, and a 0 psu salinity was measured (freshwater habitat) (Table 1). TL was measured, and the specimens were then classified into anadromous or freshwater residents based on their morphological characteristics and habitat environments, according to Higuchi and Goto^7^ and Higuchi et al.^24,25^. Overall, 144 specimens were divided into 99 anadromous and 45 freshwater residents of *G. aculeatus*, and all specimens (477) were classified as anadromous of *G. nipponicus* (Table 1).

### Otolith preparation and microchemical analysis

For a total of 671 specimens (50 specimens in the validation experiment, 621 specimens in the migration analysis), the sagittal otoliths were extracted, embedded and mounted on glass slides and were thereafter ground and polished to expose the core, as described in Arai et al.^26,27,38^. The otolith samples were cleaned in an ultrasonic bath and rinsed with deionized water prior to examination.

For electron microprobe analyses, all otoliths were Pt–Pd coated by a high vacuum evaporator. Otoliths from all specimens were used for life history transect analysis of Sr and Ca concentrations, which were measured along a line down the longest axis of each otolith from the core to the edge using a wavelength dispersive X-ray electron microprobe (JEOL JXA-8900R; JEOL, Tokyo, Japan), as described in Arai et al.^26,27,38^. Wollastonite (CaSiO_3_) and tausonite (SrTiO_3_) were used as standards. X-ray intensity maps of the Sr concentration were made using the JEOL JXA-8900R as described in Arai et al.^26,27,37^. Otoliths of 54 specimens in migration analysis were used for the life history transects that were thereafter conducted for the Sr map analyses.

### Statistical analysis

Differences between the average Sr:Ca ratios and TLs were tested by Mann Whitney-*U* tests. Differences among average Sr:Ca ratios and TLs were examined through a Kruskal–Wallis test, and subsequently post hoc Mann Whitney-*U* tests were carried out for comparisons between two groups. The significance of the correlation coefficients and regression slopes were tested by t-tests. Differences in the average Sr:Ca ratios between the low and high phases were examined through a Mann Whitney-*U* test.
